# Stubborn Contaminants: Influence of Detergents on the Purity of the Multidrug ABC Transporter BmrA

**DOI:** 10.1371/journal.pone.0114864

**Published:** 2014-12-17

**Authors:** Benjamin Wiseman, Arnaud Kilburg, Vincent Chaptal, Gina Catalina Reyes-Mejia, Jonathan Sarwan, Pierre Falson, Jean-Michel Jault

**Affiliations:** 1 Université Grenoble Alpes, Institut de Biologie Structurale (IBS), 6 rue Jules Horowitz, F-38000, Grenoble, France; 2 CEA, DSV, IBS, F-38000, Grenoble, France; 3 CNRS UMR5075, IBS, F-38000, Grenoble, France; 4 Drug Resistance Mechanism and Modulation team, Institut de Biologie et Chimie des Protéines (IBCP), UMR5086 CNRS/Université Lyon 1, 7 passage du Vercors, F-69367, Lyon, France; Zhejiang University, China

## Abstract

Despite the growing interest in membrane proteins, their crystallization remains a major challenge. In the course of a crystallographic study on the multidrug ATP-binding cassette transporter BmrA, mass spectral analyses on samples purified with six selected detergents revealed unexpected protein contamination visible for the most part on overloaded SDS-PAGE. A major contamination from the outer membrane protein OmpF was detected in purifications with Foscholine 12 (FC12) but not with Lauryldimethylamine-N-oxide (LDAO) or any of the maltose-based detergents. Consequently, in the FC12 purified BmrA, OmpF easily crystallized over BmrA in a new space group, and whose structure is reported here. We therefore devised an optimized protocol to eliminate OmpF during the FC12 purification of BmrA. On the other hand, an additional band visible at ∼110 kDa was detected in all samples purified with the maltose-based detergents. It contained AcrB that crystallized over BmrA despite its trace amounts. Highly pure BmrA preparations could be obtained using either a Δ*acrAB E. coli* strain and n-dodecyl-β-D-maltopyranoside, or a classical *E. coli* strain and lauryl maltose neopentyl glycol for the overexpression and purification, respectively. Overall our results urge to incorporate a proteomics-based purity analysis into quality control checks prior to commencing crystallization assays of membrane proteins that are notoriously arduous to crystallize. Moreover, the strategies developed here to selectively eliminate obstinate contaminants should be applicable to the purification of other membrane proteins overexpressed in *E. coli*.

## Introduction

Membrane proteins play a pivotal role in the cells as they orchestrate the exchange of information, nutrients or waste with the external milieu. They constitute about one third of the proteome in each living organism, and given their vital role, they are targeted by the majority of the drugs currently on the market [1]. Yet, despite recent advances in protein expression and purification technologies, they remain one of the most difficult groups of proteins to study. Crystallization in particular remains a challenging endeavor and while some success has been achieved recently [2], [3], producing sufficient quantities of highly pure and homogenous samples suitable for crystallization continues to be a major stumbling block for most membrane proteins. Choosing the correct detergent for solubilization and purification is integral to this process. A search of the Membrane Protein Data Bank revealed that n-dodecyl-β-D-maltopyranoside (DDM), n-dodecyl-N,N-dimethylamine-N-oxide (LDAO), and octyl-β-D-glucopyranside (OG) have been the three detergents most successfully used for the solubilization, purification, and crystallization of membrane proteins [4]. Although these detergents are a good starting point, typically many detergents need to be screened before the best ones are chosen for a protein of interest. Detergent screens are normally accomplished by various quality control tests including functional assays, monitoring the sharpness and symmetry of the SEC profile, and purity by SDS-PAGE and only the detergents that produce highly pure and monodispersed samples are kept for further crystallization trails [5]. In this way many detergents can be quickly screened in a high-throughput manner ensuring that only the best samples with the highest probability of crystallization are used.

Obtaining pure protein is key to this process. Purity is usually assessed simply by visualization on SDS-PAGE and as a result many contaminating proteins can be potentially overlooked either by migrating at the size of the target protein or being present as trace amounts not easily spotted. There have been several reports of minute contaminants being accidentally crystallized during initial screening for crystallization conditions [6]-[12], resulting in the waste of time and resources, pursuing initially encouraging but ultimately false leads. Typically, analyses of purified protein for contaminants are only conducted after attempts at structure determination using models of the target protein have failed. Incorporating a proteomics-based purity analysis into quality control checks [13], prior to commencing crystallization trails can be a fast and cost effective method for minimizing the crystallization and time wasted on structure determination of contaminants. Although, crystallization is in itself a purification step and trace contaminants will always be present, purifications with too many contaminants can be reassessed with improved protocols before commencing crystallization trials. At the very least if the identity of the contaminants are already known, they can be quickly checked by SDS-PAGE against any new diffracting crystals.

Recently the use of fluorescence size-exclusion chromatography (FSEC) [14], [15] and multicolor fluorescence size-exclusion chromatography (MC-FSEC) [16], has created the ability to screen detergents and other buffer parameters in a high-throughput manner allowing scientists to screen potentially hundreds of combinations in a fraction of the time compared to traditional SEC methods. With the use of fluorescence tags such as the green fluorescent protein (GFP) one can monitor the location, expression levels, monodispersity, approximate molar mass [15] and thermal stability [17] of the target membrane protein using nanogram or less quantities of either purified or unpurified protein. However since these methods only require nanograms or less of the target protein compared to milligrams or micrograms using traditional SEC methods trace contaminates that could interfere or crystallize over the target protein might be easily overlooked.

BmrA (Bacillus multidrug resistance ATP) is a *Bacillus subtilis* multidrug transporter that belongs to one of the largest families of membrane proteins known as the ATP-binding cassette (ABC) proteins. In this family, membrane transporters are responsible for either the uptake or export of many different substrates across membranes. Among exporters, some efflux a wide array of molecules including anti-cancer drugs and antibiotics implicating them in multidrug resistance (MDR) phenotypes in human cancers cells and pathogenic bacteria, respectively [18]. ABC transporters share a common core architecture consisting of two soluble nucleotide-binding domains and two transmembrane domains that couple ATP binding and hydrolysis with the transport of molecules across a membrane and against a concentration gradient [19]. Despite several reported structures of exporters [20]-[24] many questions remain unanswered about the exact mechanism of substrate recognition and drug efflux.

Producing highly pure protein suitable for crystallization trials is the first step in attempting to solve new high-resolution structures of any protein. Because BmrA can be expressed in large amounts in *E. coli* [25] and is easily purified in a functional state [26], this makes this transporter a suitable target for structural approaches. First, given the high yield of solubilized protein, the detergent FC12 was initially chosen to purify and crystallize BmrA. This unfortunately led to the crystallization of the contaminant OmpF in a new space group that is reported here. Therefore, we selected six further detergents to study the solubilization and purity of the extracted BmrA. The homogeneity of the seemingly purified transporter preparations was analyzed by mass spectrometry for each detergent used. Despite a standard two-step purification protocol involving nickel affinity and size exclusion chromatographies, multiple contaminants were still detected, although present in trace amounts for most of them. The use of maltose-based detergent to purify BmrA allowed us to avoid the contamination by OmpF but AcrB was present as a minor contaminant and thus crystallize instead of BmrA. In this report, strategies to overcome these unfortunate scenarios are proposed that could be directly applicable to other membrane proteins overproduced in *E. coli* for functional and/or structural purposes.

## Materials and Methods

### Expression and solubilization of BmrA with different classes of detergent

BmrA was cloned with a 6-histidine *N*-terminal tag, overexpressed in C41(DE3) *E. coli*, unless otherwise specified, and membrane fraction containing overexpressed BmrA was prepared as previously described [25]. A set of non-ionic and zwitterionic detergents were chosen to determine which type of detergent was most effective for BmrA extraction. Solubilization screen was conducted in 500 µL total volume mixtures with membranes diluted to 5 mg/mL and solubilized at a detergent concentration of 1% (w/v) in buffer containing 50 mM Tris-HCl pH 8.0, 100 mM NaCl and anti-proteases tablets (Roche), with the following detergents: n-dodecyl-β-D-maltopyranoside (DDM), n-undecyl-β-D-maltoside (UDM), n-undecyl-β-D-thiomaltoside (UTM), lauryl maltose neopentyl glycol (LMNG), decyl maltose neopentyl glycol (DMNG), octyl β-D-glucopyranoside (OG) 6-cyclohexyl-1-hexyl-β-D-maltoside (Cymal 6), Lauryldimethylamine oxide (LDAO) and zwitterionic n-dodeclyphosphocholine (FC12), and n-Hexadecylphosphocholine (FC16). After incubation for 2 h at 4°C, soluble and insoluble fractions were separated by centrifugation at 180,000 g for 60 min. Insoluble material was suspended in buffer and equivalent protein concentrations were analyzed by 10% SDS-PAGE.

### Purification of BmrA in FC12

Membranes containing overexpressed BmrA were solubilized at 2 mg/mL by gentle mixing in 50 mM Tris-HCl pH 8.0, 100 mM NaCl, 0.1 mM DTT, 0.02 mM EDTA and anti-protease tablets (Roche) with 1% (w/v) of FC12 for 2 h at 4°C. Insoluble material was removed by ultracentrifugation at 100,000 g for 1 h at 4°C. The supernatant containing the solubilized BmrA was diluted 2 times with buffer A (50 mM Tris-HCl pH 8.0, 0.5% FC12 and anti-protease tablets) and loaded at a flow rate of 2 mL/min onto to a 5 mL pre-equilibrated Q-Sepharose ion exchange column (*GE Healthcare*). After washing with 10 mL of buffer A, proteins were eluted by a gradient from 0 to 100% with 50 mL of 50 mM Tris-HCl pH 8.0, 1 M NaCl, 0.5% FC12 and fractions of 1 mL were collected. The fractions containing BmrA were pooled and loaded onto a 5 mL HiTrap chelating HP column (*GE Healthcare*) pre-equilibrated with buffer B (50 mM Tris-HCl pH 8.0, 200 mM NaCl, 10 mM imidazole, 0.3% FC12 and anti-protease tablets). After washing with 50 mL of buffer B, the elution was made by a linear gradient from 0 to 100% with 30 mL of buffer C (50 mM Tris-HCl pH 8.0, 200 mM NaCl, 500 mM imidazole, 0.3% FC12 and anti-proteases tablets). The eluted fractions containing BmrA were pooled and dialysed overnight at 4°C, using a 50 kDa cut-off, and against 1 L of buffer containing 50 mM Tris-HCl pH 8.0, 100 mM NaCl and 0.15% FC12. Finally, BmrA was concentrated to 20 mg/mL by centrifugation at 4,000 g on an Amicon ultrafiltration device (MWCO 50 kDa, Millipore).

### Purification of BmrA with a set of six selected detergents

When BmrA was solubilized from the membranes and purified with a set of six selected detergents (DDM, UDM, UTM, LMNG, FC12, and LDAO) all purification steps were carried out at 4°C. Membranes containing overexpressed BmrA were solubilized with 1% of each detergent with gentle mixing for 90 min in 50 mM Tris-HCl pH 8.0, 100 mM NaCl, 10% glycerol, 10 mM imidazole, 1 mM PMFS, and anti-protease tablets (Roche). Following centrifugation at 100,000 g for 60 min to remove non-solubilized proteins, the supernatant was incubated with gentle mixing for 60 min with 1-2 mL of equilibrated Ni^2+^–NTA resin. Bound protein was washed with 100 volumes of 50 mM Tris-HCl pH 8.0, 10% glycerol, 100 mM NaCl, 20 mM imidazole, 1 mM PMSF, EDTA-free anti-protease tablets and detergent (0.05% DDM, 0.2% UDM, 0.05% UTM, 0.02% LMNG, 0.2% LDAO, or 0.3% FC12). After washing, bound protein was eluted from the Ni^2+^–NTA resin with 2–3 volumes of 250 mM imidazole in the same buffer. Finally, BmrA was purified by SEC/FPLC on a HiLoad 16/60 Superdex 200 PG column (GE Healthcare) equilibrated with 25 mM Tris-HCl pH 8.0, 150 mM NaCl, and detergent (0.02% DDM, 0.1% UDM, 0.03% UTM, 0.005% LMNG, 0.1% LDAO, or 0.15% FC12). Fractions corresponding to BmrA dimers were pooled and concentrated using a 100 kDa MWCO filter (Millipore), flash frozen in 100 µL aliquots in liquid nitrogen and kept at -80°C, if not used immediately for analysis.

### Mass spectrometry analyses of fractions containing purified BmrA

The purity of BmrA samples was analyzed by overloading a 10% SDS-PAGE in order to detect the presence of contaminants in addition to the protein of interest. Samples were incubated at 37°C and approximately 15 µg of purified protein were resolved on SDS-PAGE gels and visualized with Coomassie Brilliant Blue. Visible bands were cut out and protein(s) within these bands were identified from their fragments generated by in-gel trypsin digestion followed by MALDI-TOF mass spectrometry [13].

### Crystallization and structure resolution

Crystallization trials were carried out using the crystallogenesis platform at the IBCP or the High Throughput Crystallization Laboratory at the EMBL Grenoble [27], using the vapor diffusion technique on hanging drops over 96 well plates to search for initial hits. Crystals were then manually optimized in 24 well plates. Final optimized conditions were as follows: BmrA at 10 mg/mL, incubated with 5 mM ADP for 30 min on ice and mixed in a 1∶1 ratio with a reservoir solution containing 18–22% PEG 1500, 5–10% 2-Methyl-2,4-pentanediol and 0.1 M Tris-HCl pH 8.0 at 19°C. Crystals appeared overnight with a high variation in nucleation profiles. Large crystals were harvested, cryo-protected by a quick dip in a solution containing the well solution supplemented by 10% glycerol, and flash cooled in liquid nitrogen.

Diffraction screening was performed on ID23-1 and ID14 beamlines at ESRF and Proxima-1 at SOLEIL, respectively, and data were collected on beamline ID23-1 at ESRF. Data came from a single rod-shaped crystal shot at two distinct locations and merged. Data was processed using Mosflm [28] and scaled using Aimless [29]. Phases were obtained by molecular replacement using Phaser [30] in the phenix 1.8.1-1168 package [31] to use the latest version able to handle translational pseudosymmetry, using a trimer of OmpF as a search model (PDB: 2ZFG). The structure was refined using phenix.refine [32] using restrained refinement with group B refinement (two groups per residue) and TLS (1 TLS group per chain), and manual rebuilding in Coot [33]. Non-crystallographic symmetry was used throughout the refinement.

## Results and Discussion

### Extraction of BmrA using a panel of detergents

**Figure 1:**
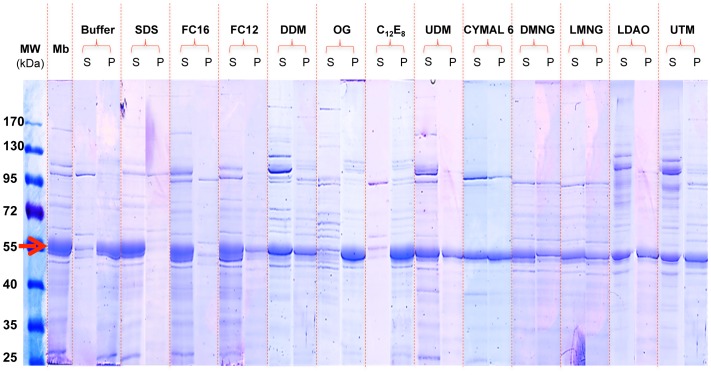
Extraction of BmrA in *E. coli* membrane with 1% (w/v) detergents. After solubilization with the indicated detergent, extracted and non-extracted materials were separated by ultracentrifugation, and the supernatant (S) and pellet (P) were resolved on a 10% SDS-PAGE. Fifteen μl of soluble and insoluble fractions were loaded (∼75 µg of protein). Positive control experiment was carried out with sodium dodecyl sulfate (SDS) and negative control was carried out without detergent (buffer alone). Mb: the membrane fraction. Red arrow indicates the position of BmrA.

The choice of a detergent for solubilization of a membrane protein is a critical parameter for its subsequent purification, and although previous studies have shown that BmrA can be purified in a functional state by using DDM [26], [34]–[36], we have revisited this step to ascertain whether this detergent is the most appropriate to get the highest possible yield of BmrA in a state as pure and stable as possible. Thus, we compared the extraction efficiencies of BmrA using several non-ionic detergents: DDM, UDM, LMNG, DMNG, OG, C_12_E_8_ and Cymal 6, and zwitterionic detergents: FC12 and FC16; SDS was used as a positive control for a maximal extraction efficiency (Fig. 1). As expected, SDS was found to extract the largest amount of protein from the membrane while OG and C_12_E_8_ were poorly efficient, leading to a very low recovery of BmrA in the supernatant similar to that found in the sample incubated with the buffer only. For OG, this result contrasts with many *E. coli* membrane proteins that are normally efficiently solubilized by this particular detergent [37]. The lipid-like surfactants FC12 and FC16 were almost as good as SDS with ∼90% of BmrA being solubilized. In contrast, DDM and the other sugar-based detergents extracted less than half of the total protein. Several examples in the literature like LmrA [38], BmrC/D [39], ABCG2 [40], ABCB4 and ABCB11 [41], and ABCA4 [42] have shown the preference of ABC transporters, or membrane proteins in general [37], for foscholine detergents in solubilization screens. Thus, based on its high efficacy in this solubilization screen, we chose to pursue with FC12 as the detergent of choice for purification of BmrA for structural studies.

### Purification of BmrA in FC12

**Figure 2:**
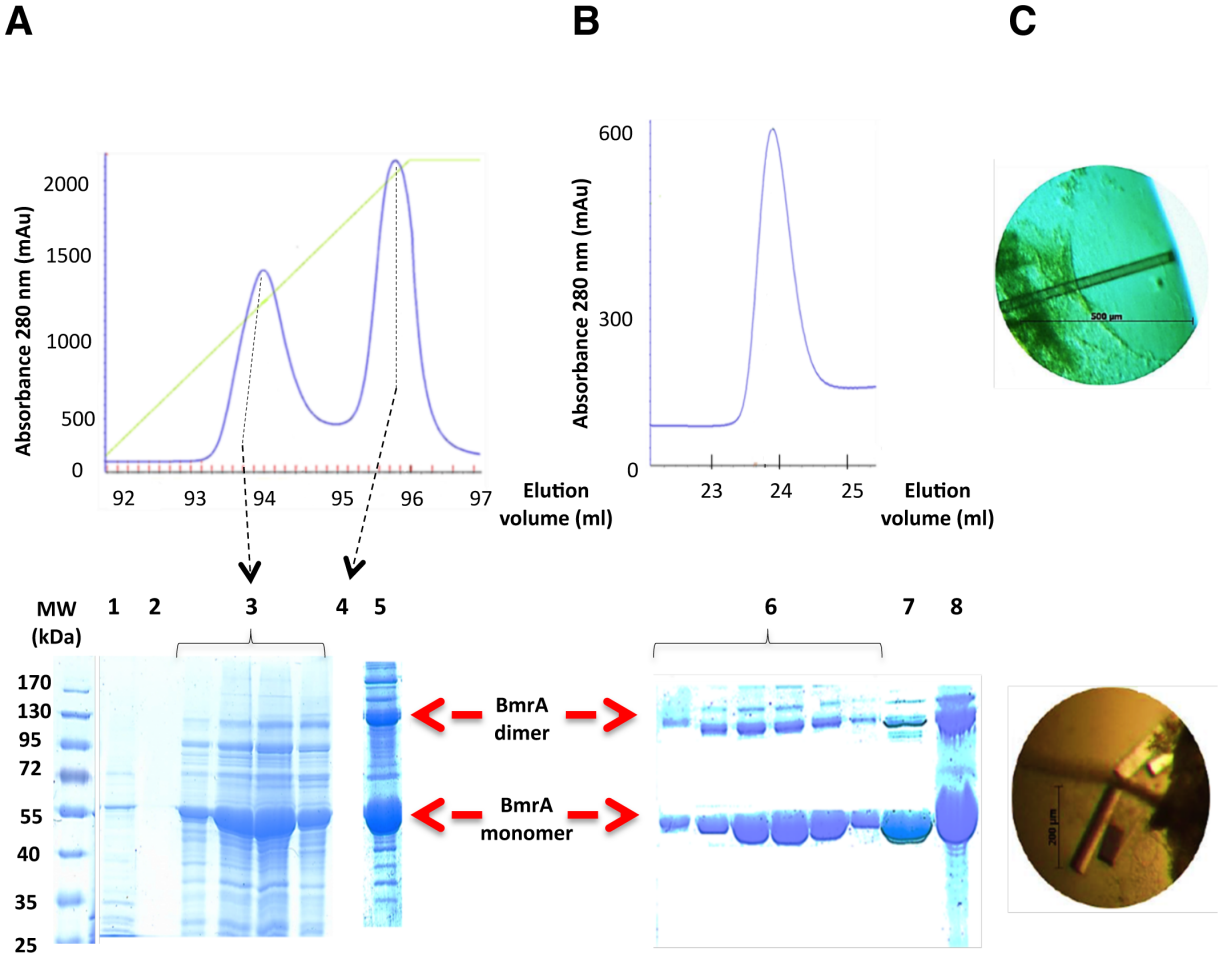
Purification and crystallization of BmrA protein in FC12. ***A***, elution of FC12 solubilized BmrA protein from a Q sepharose fast flow column. The absorbance of the protein was monitored at 280 nm and BmrA was eluted from the column with 600 mM NaCl. BmrA fractions were resolved on 10% SDS-PAGE. Lane 1, loading step. Lane 2, washing step. Lanes 3 and 4, protein eluted during the NaCl gradient. Lane 5, pool. Red arrows indicate the positions of the BmrA dimer and monomer. ***B***, elution of BmrA from a Ni^2+^ high trap chelating column. SDS-PAGE, Lane 6, peak fractions. Lane 7, pool. Lane 8, concentrated pool. ***C***, crystals obtained at 20°C by the vapor-diffusion hanging drop method with 2 µl of 10 mg/ml purified BmrA and 2 µl of the reservoir solution (18% polyethylene glycol 1500, 10% 2-methyl-2,4-pentanediol and 100 mM Tris-HCl pH 8.0).

BmrA was extracted from the membranes with 1% FC12 and loaded onto an anion exchange resin. After a washing step, proteins were eluted from the column with the addition of 0.6 M NaCl (Fig. 2***A***). BmrA was found in the first peak, while the second peak most likely contained nucleotides based on the high 260/280 ratio. The band observed at approximately 130 kDa corresponds to BmrA dimers that are resistant to denaturing conditions of SDS-PAGE. Fractions containing BmrA were then pooled, loaded onto a Ni^2+^-NTA agarose column and BmrA was eluted with the addition of 0.5 M imidazole (Fig. 2***B***). The chromatogram and corresponding SDS-PAGE showed that BmrA was eluted in a homogeneous peak, thin and symmetrical, that still contains some SDS-resistant BmrA dimers. Peak fractions were pooled, dialyzed to reduce the concentration of FC12 to 0.15%, and then concentrated to 20 mg/mL. The concentration of FC12 was quantified to 3.5% by a method developed in our laboratory (Chaptal *et al.,* in preparation). This purification protocol yielded approximately 8 mg of BmrA from five liters of culture.

### Crystallization of the purified protein

**Figure 3:**
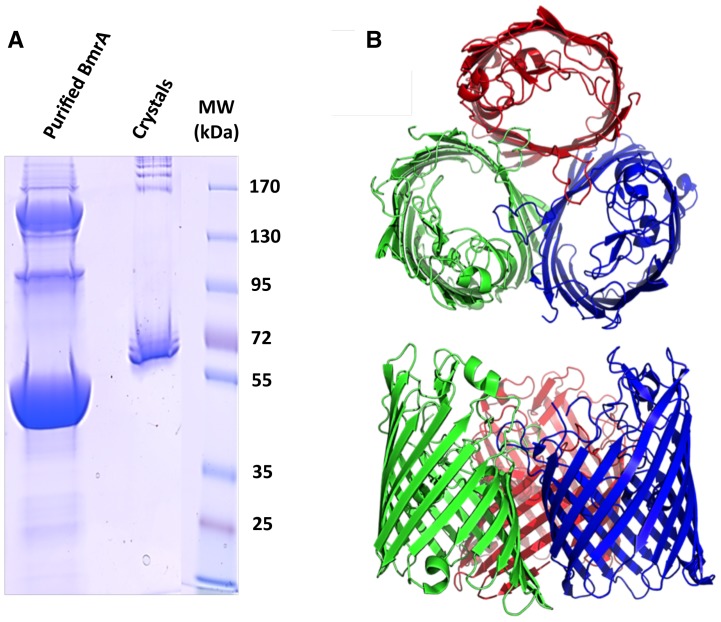
Crystallization of OmpF in the detergent FC12 purified BmrA sample. ***A***, purified BmrA in FC12 and 25 washed OmpF crystals were resolved on a 10% SDS-PAGE. ***B***, crystal structure of an OmpF trimer in two orientations related by a 90° rotation (each monomer is shown in different colors).

Initial screens carried out using the PEG I and II suites led to several positive hits appearing in one week. The best one led to colorless rod shaped crystals reaching about 1.0×0.2×0.1 mm in size, and were obtained for BmrA that was co-crystallized with 5 mM ADP, which diffracted to 3.8Å (Fig. 2***C***, S1 Table). Surprisingly, molecular replacement attempts with structures of different ABC exporters did not lead to any solution despite the wealth of different conformations available. To assess the presence of BmrA within the crystals, we harvested a large number of crystals after careful washing in several drops of crystallization solution, and identified their content on SDS-PAGE (Fig. 3***A***). A band migrating above the apparent molecular mass of BmrA was observed, and sequencing by mass spectrometry unambiguously revealed that the protein present in this band was in fact OmpF (S2 Table).

OmpF migrated with an apparent unusual size, ∼60–70 kDa, while the protein was expected to migrate at ∼40 kDa. In addition, the corresponding band of OmpF was not observed in the BmrA purified fraction (Lane 1). This result led us to analyze more carefully the other bands of the purified fraction (see below). Once OmpF was identified in the crystals, the molecular replacement gave a solution. Crystals belonged to the monoclinic space group *C* 2 and contained two trimers in the asymmetric unit. The diffraction data sets were refined to 3.8 Å (S1 Table).

### Purification of BmrA with six selected detergents

**Figure 4:**
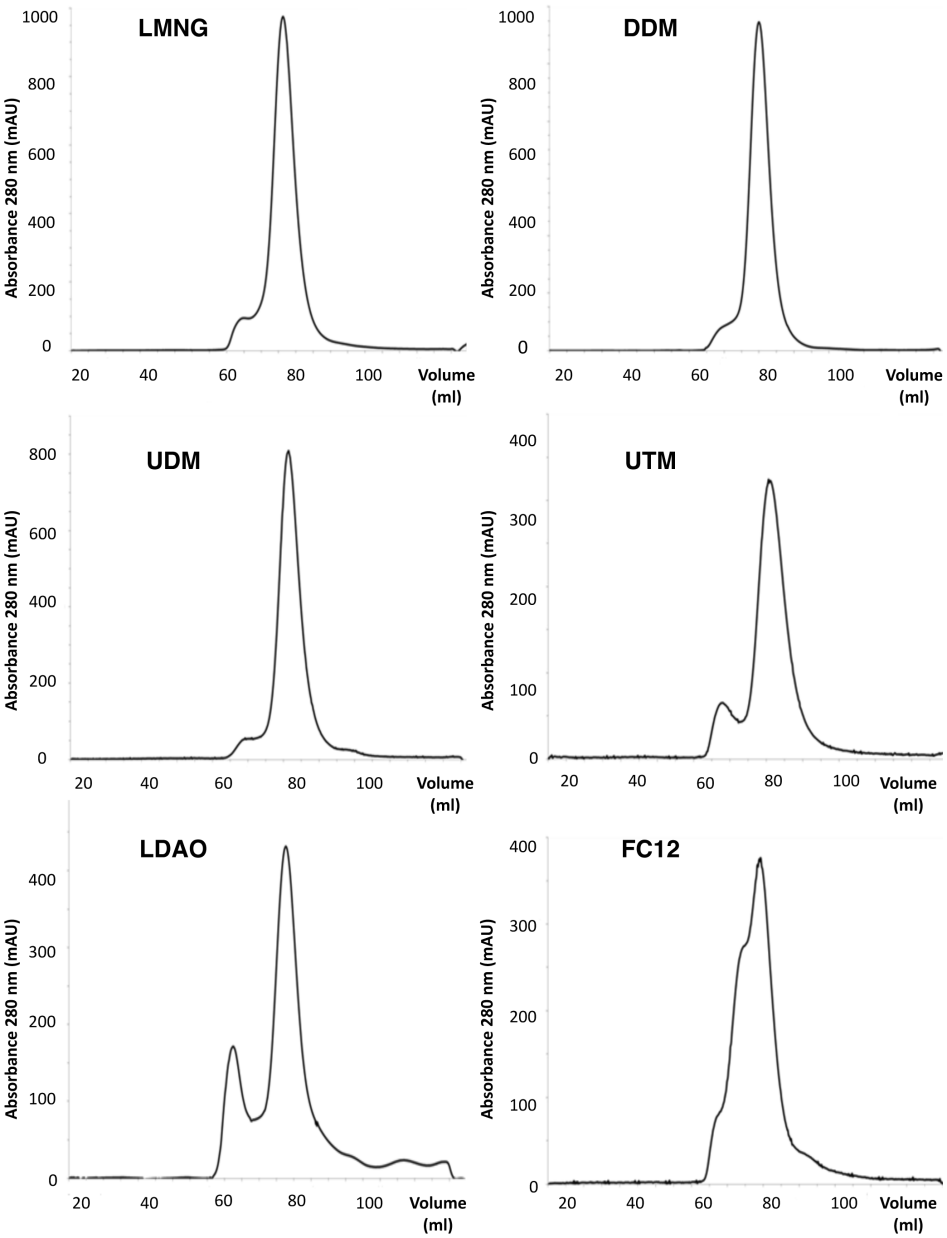
Size exclusion chromatography profiles of BmrA purified in six selected detergents. BmrA was solubilized from *E. coli* membrane and purified with the detergents as indicated in the figure.

Given the propensity of FC12 to co-extract and co-purify OmpF with BmrA, we re-examined our choice of the detergent used for the purification. From the solubilisation screen (Fig. 1), several maltose-based detergents (i.e. DDM, UDM, UTM and LMNG) plus LDAO were selected for further study and FC12 was kept as a control due to its high efficacy of extraction. After solubilisation and nickel affinity chromatography, BmrA containing fractions were pooled and analyzed by gel filtration. It should be noted that in the construct used here BmrA contained a His-tag fused at its *N*-terminus whereas previous purification protocols were carried out with a His-tag fused at the *C*-terminus of the protein [26], [34], [35], [43]. This, however, does not seem to impact neither on the expression yield nor on the retention of the protein on the nickel column; this latter property shows that the affinity tag is equally accessible in both constructs. Samples solubilized and purified in DDM, UDM, UTM, FC12, LDAO, and LMNG all eluted from the gel filtration column at the volume corresponding to the expected elution time for BmrA dimers in detergent micelles (Fig. 4., peaks at ∼60–62 mL), consistent with previous studies of BmrA purified in DDM [43] and two related bacterial transporters also purified in DDM [39], [44]. The four maltose-based detergents, LMNG, DDM, UDM, and UTM, all yielded a similar amount of aggregation as seen by a small shoulder at approximately 50 mL with the maximum of ∼10% in UTM. In LDAO, dimers of BmrA had a slightly retarded elution of 62±0.5 mL consistent with a smaller micelle size [45] and generally higher aggregation of about 30%. Purifications with FC12 on the other hand yielded a broader SEC profile with two distinct shoulders at about 47 mL, 55 mL, and the maximum at 60±0.5 mL.

**Figure 5:**
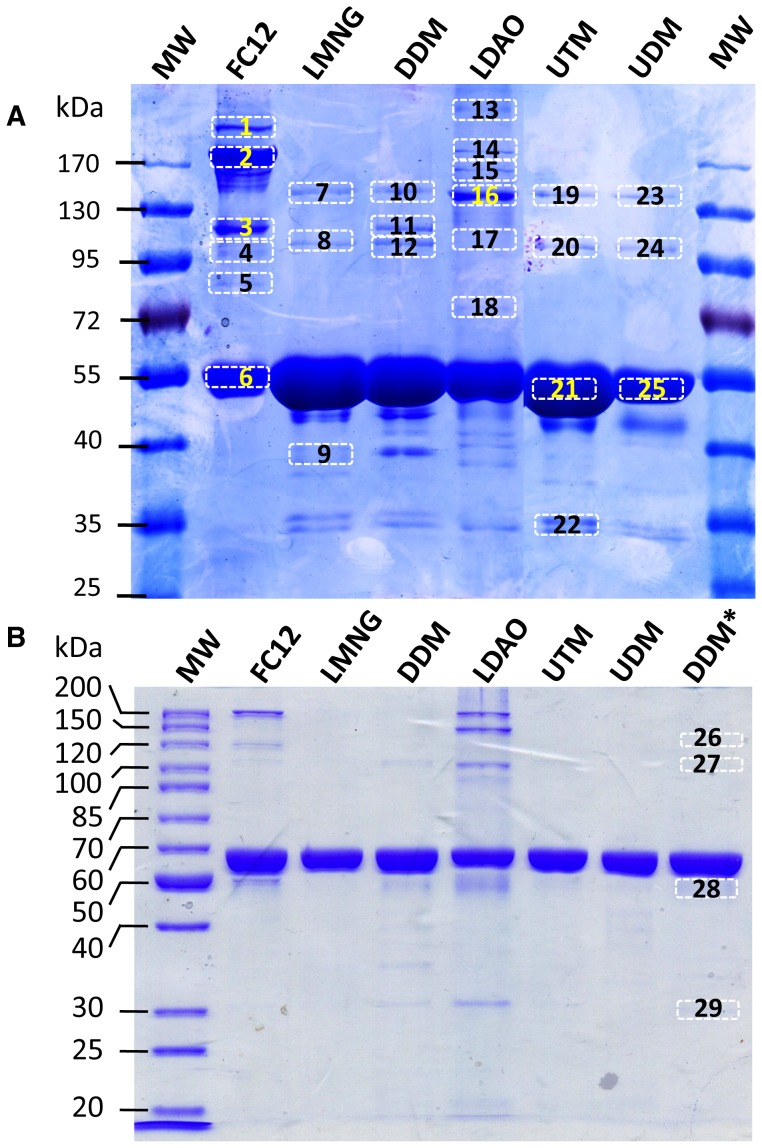
SDS-PAGE of the purified BmrA. ***A***, 10% SDS-PAGE of 15 µg of purified BmrA in the detergents as indicated in the figure. The numbered bands were cut out and their trypsin digested products were analyzed by LC-MS/MS (see table 1). ***B***, 10% SDS-PAGE of 5 µg of purified BmrA in the detergents as indicated in the figure. *BL21(DE3)Δ*acrAB*, Δ*acrEF* was used for the overexpression of BmrA. Please note that samples for FC12 in panels ***A*** and ***B*** were obtained from two different purifications protocols (see the text).

In order to detect the possible contaminants that co-purify with BmrA, samples obtained from each of the six purification protocols were deliberately overloaded (15–20 µg of proteins) and resolved by SDS-PAGE (Fig. 5***A***). Indeed, a gel performed in a standard way with 5 µg of proteins loaded (Fig. 5***B***) revealed a seemingly pure BmrA preparation for all detergents tested but FC12 and LDAO and to a lesser extent DDM. A predominant band with an apparent molecular mass of ∼55 kDa and corresponding to BmrA was observed in all cases but for the FC12 purification where additional bands of higher apparent molecular weights were also clearly visible (Fig. 5***A***). The four maltose-based detergents LMNG, DDM, UDM, and UTM also had similar contamination-like patterns with additional bands at approximately 150, 110, 45 and 34 kDa and in the case of DDM extra bands at 115 and 40 kDa (Fig. 5***A***). The ‘contaminating’ and/or degradation bands in the maltose-based detergents appear negligible as compared to the main band of BmrA representing probably no more than a few percents in total (Fig. 5***B***). Purifications in LDAO contained the same two bands at 110 and 150 kDa seen in the maltosides, and four other weaker bands. LDAO purifications also did not contain the same pattern below the BmrA band at 55 kDa with only a single band visible at 34 kDa compared to two with the maltoside-based detergents, and no visible band directly below the BmrA band at 45 kDa. Purifications in FC12 displayed a unique pattern compared to the other detergents and does not have any bands in common other than the main BmrA band. Notably, the three top bands are in a much higher ratio to the BmrA band at approximately 10, 70, and 20% respectively. Interestingly, there were no visible bands below the main BmrA band in purifications in FC12. It is important to note here that the two FC12 samples analyzed in panels ***A*** and ***B*** (Fig. 5) came from two different purifications protocols. In panel ***A***, the sample was the one used for crystallization trials and was subjected to two columns, DEAE plus Ni-agarose, before being concentrated up to 20 mg/mL whereas sample from panel ***B*** was treated as for the other detergents with a Ni-agarose column followed by a gel filtration. This is presumably why less BmrA ‘oligomeric forms’ were detected in panel ***B*** (see below).

### Impurity identification by mass spectrometry

Table 1 summarizes the detected ‘contaminants’ in the six detergent purified samples. Western blot analysis revealed that the majority of the bands were either aggregates and/or oligomeric forms of BmrA in the case of the bands above the 55 kDa band or degraded products in the case of the bands below the 55 kDa band (not shown). However, further analysis with mass spectrometry revealed that many of the additional bands contained multiple proteins along with BmrA. For example, the bands at 45 and 40 kDa (bands 14 and 15) in the DDM sample contained 11 and 5 additional detectable proteins, respectively, although BmrA appears to be present in large excess in these two bands. Also, while the main bands at 55 kDa of UTM and UDM were pure, the upper bands contained 4 and 8 contaminating proteins, respectively. It is interesting to note that for the undecyl-maltosides all the contaminants detected in UTM were also seen in UDM. And aside from the well-known contaminant acriflavine resistance protein B (AcrB), which was detected in all the maltoside samples, purifications with DDM only had ubiquinol oxidase and undecaprenyl-phosphate 4-deoxy-4-formamido-L-arabinose transferase in common with UTM and UDM.

**Table 1:** Impurity identification of purified BmrA in the various detergents.

Detergent	Band^a^	Score	# of Peptides	Coverage (%)	Mw (kDa)	Accession #	Name
**FC 12**	1 (230 kDa)	2 204	59	21	64.5	O06967	**BmrA**
		361	13	15	39.3	P02931	Outer membrane protein F (OmpF)
	2 (180 kDa)	19 119	402	44	64.5	O06967	**BmrA**
		246	4	7	39.3	P02931	OmpF
	3 (120 kDa)	14 785	442	44	64.5	O06967	**BmrA**
		6 760	235	53	39.3	P02931	OmpF
	4 (110 kDa)	510	12	18	64.5	O06967	**BmrA**
		217	7	15	39.3	P02931	OmpF
	5 (85 kDa)	591	19	19	82.1	P05825	Ferrienterobactin receptor (fepA)
		306	9	13	64.5	O06967	**BmrA**
		116	3	8	39.3	P02931	OmpF
	6 (55 kDa)	9 119	209	60	64.5	O06967	**BmrA**
		2 164	63	52	82.1	P05825	fepA
		1 163	34	57	39.3	P02931	OmpF
**LMNG**	7 (150 kDa)	429	14	22	64.5	O06967	**BmrA**
	8 (110 kDa)	196	4	3	113.5	P31224	Acriflavine resistance protein B (AcrB)
	9 (45 kDa)	188	5	10	64.5	O06967	**BmrA**
**DDM**	10 (150 kDa)	108	2	5	64.5	O06967	**BmrA**
	11 (120 kDa)	185	3	5	64.5	O06967	**BmrA**
	12 (110 kDa)	418	8	7	113.5	P31224	AcrB
		114	2	5	64.5	O06967	**BmrA**
**LDAO**	13 (250 kDa)	327	14	13	64.5	O06967	**BmrA**
	14 (180 kDa)	596	18	20	64.5	O06967	**BmrA**
	15 (170 kDa)	466	11	13	64.5	O06967	**BmrA**
	16 (150 kDa)	18 727	475	50	64.5	O06967	**BmrA**
		2 428	70	26	34.9	P0ABJ2	Cytochrome ubiquinol oxidase sub. 2
		297	5	6	74.3	P0ABJ0	Cytochrome ubiquinol oxidase sub. 1
	17 (110 kDa)	104	3	5	64.5	O06967	**BmrA**
	18 (75 kDa)	n.d.	n.d.	n.d.	n.d.	n.d.	n.d.
**UTM**	19 (150 kDa)	n.d.	n.d.	n.d.	n.d.	n.d.	n.d.
	20 (110 kDa)	1 836	44	21	113.5	P31224	AcrB
		421	11	8	99.6	P0AFG9	Pyruvate dehydrogenase E1 component
		222	4	3	114.6	Q8FK36	Cation efflux system protein (CusA)
		158	3	3	105.0	P0AFG5	2-oxoglutarate dehydrogenase E1
	21 (55 kDa)	15 230	427	57.2	64.5	O06967	**BmrA**
	22 (35 kDa)	227	5	16	36.3	A7ZP72	Undecaprenyl-phosphate 4-deoxy-4-formamido-L-arabinose transferase
**UDM**	23 (150 kDa)	177	8	7	64.5	O06967	**BmrA**
		102	2	13	34.9	P0ABJ2	Cytochrome ubiquinol oxidase sub. 2
		95	2	8	36.3	A7ZP72	Undecaprenyl-phospate 4-deoxy-4-formamido-L-arabinose transferase
	24 (110 kDa)	3 204	65	19	113.5	P31224	AcrB
		1 574	51	26	99.6	P0AFG9	Pyruvate dehydrogenase E1
		638	19	11	105	P0AFG5	2-oxoglutarate dehydrogenase E1
		351	9	9	64.5	O06967	**BmrA**
		254	6	5	102.0	A7ZHI9	Protein translocase subunit (SecA)
		158	3	3	99.4	P0ABB9	Magnesium-transporting ATPase
		120	4	4	114.6	Q8FK36	CusA
	25 (55 kDa)	34 422	903	61	64.5	O06967	**BmrA**
DDM*	26 (150 kDa)	n.d.	n.d.	n.d.	n.d.	n.d.	n.d.
	27 (110 kDa)	95	5	4	99.6	P0AFG9	Pyruvate dehydrogenase E1
		90	2	2	105	P0AFG5	2-oxoglutarate dehydrogenase E1
	28 (50 kDa)	n.d.	n.d.	n.d.	n.d.	n.d.	n.d.

a band numbering correspond to the bands cut out from the 10% SDS-PAGE in Fig. 5. The apparent molecular weights as estimated by migration on the gel are indicated in parenthesis. n.d., no protein detected. The score is a protein quality identification index, considering the number of peptide sequences and MS/MS spectra that have been identified for each protein.

Purification with LMNG was remarkably pure with only AcrB being detected in the 110 kDa band. The detection of BmrA in the 150 kDa band is consistent with SDS-resistant dimers. Its detection in the other bands at 110 kDa and 120 kDa in DDM and UDM suggests a small amount of aggregation and/or some degradation of BmrA dimers.

Purification in FC12 contained qualitatively few contaminating proteins when compared to purifications in the other detergents, except LMNG, although the outer membrane porin OmpF was detected in all analyzed bands. In addition the outer membrane receptor ferrienterobactin was detected in the main BmrA band at 55 kDa and band 5 at 85 kDa. Detection in band 5 is consistent with the expected mass of Ferrienterobactin of 82.1 kDa; however its detection in the main BmrA band at 55 kDa suggests some degradation and/or non-specific interactions with BmrA.

The multiple bands of OmpF and BmrA observed in FC12 suggests aggregation and/or non-specific interaction and is consistent with the non-monodispersed SEC profile (Fig. 5), especially the large band at 180 kDa (band 2) that is roughly 70% of the main BmrA band. It is interesting to note here that OmpF contamination overlaps the migration pattern of BmrA perfectly. Unfortunately, the intense band at 120 kDa (band 3) with roughly 20% of the total intensity of the main BmrA band is consistent with both a BmrA dimer and an OmpF trimer making its initial detection difficult. Porins are known to be extremely stable proteins with the trimer able to withstand denaturation with 5 M guanidium hydrochloride or 2% SDS at 70°C [46]. Thus, perhaps unsurprisingly, there is no visible band corresponding to an OmpF monomer, even on overloaded gels (Fig. 5***A***), that might have hinted at porin contamination. Indeed the strongest OmpF score of 10297 is observed in band 3 consistent with the OmpF trimer. The main BmrA band at 55 kDa also contains a relatively strong score of 1163 that could be consistent with an OmpF dimer, and even washed crystals migrated in a way consistent with an SDS resistant dimer (Fig. 3), albeit at a slightly larger size than the main BmrA band and despite attempts to denature the protein with 2% SDS. Due to the symmetry of the OmpF trimer [46], why a SDS resistant dimer would persist over the trimer is not readily obvious, although this might be due to some aberrant migration behavior of the trimer in SDS-PAGE.

Finally, purifications with LDAO contained 6 additional contaminating proteins. Notably, however, both AcrB and OmpF are absent. Similar to FC12, all analyzed bands in LDAO contained BmrA suggesting non-specific interactions with contaminating proteins. Unlike FC12, purifications in LDAO have a symmetrical mono-dispersed SEC profile similar to maltoside-based detergents (Fig. 4). Yet, a larger peak is observed in the void volume of the column as compared to maltoside-based detergents suggesting that BmrA is less stable in LDAO and aggregates over time. This is consistent with the smaller micelle size of LDAO that would potentially favor crystal contacts but also nonspecific aggregation. Like in the maltoside solubilized samples, there does not seem to be one overwhelming contaminant, although there is a particularly strong score of 2428 for the respiratory enzyme ubiquinol oxidase in the 150 kDa band (Fig. 5, band 16). Ubiquinol oxidase has being previously crystallized in octyl-β-D-glucopyranoside [47] and there are no reports of its accidental purification, although its presence along with the other contaminants would undoubtedly interfere with the crystallization of the target protein.

### How to get rid of OmpF and AcrB?

It is interesting that there are only two contaminating proteins in FC12 compared with nine in DDM. Since FC12 is one of the most effective membrane solubilizers [10], one might have expected to find more contaminating proteins when compared to DDM and other maltose-based detergents that are known to be less effective, but this is not the case. Possibly, the lipid-like zwitterionic nature of FC12 is more effective at disrupting nonspecific interactions of other contaminating proteins with the Ni^2+^-NTA resin resulting in a cleaner purification. On the other hand, because FC12 solubilizes almost all BmrA present in the membrane in contrast to maltoside-based detergents that are less efficient, this might overwhelm the presence of contaminants in the purified samples.

Due to a histidine-rich cluster at its *C*-terminus AcrB is a well-documented contaminant in membrane protein purifications using Ni^2+^-NTA resins in their purifications steps and has repeatedly been crystallized by accident [6], [7], [9]. In this study, AcrB was detected only in the maltoside-based detergent purified samples. And as a matter of fact, when DDM was used for the purification of BmrA, we did obtained crystals of proteins which turned out to be AcrB (not shown). Although there is no report of AcrB being crystallized with FC12, it is still surprising that it was absent considering the efficiency of FC12 membrane solubilization as well as the known affinity of AcrB with Ni^2+^-NTA. Since it is unlikely that AcrB is not solubilized with FC12, a possible explanation for its absence in the purified samples is that FC12 is acting somehow to disrupt its non-specific binding to the Ni^2+^-NTA. In all 26 reported structures of AcrB the detergent used for crystallization was a maltoside derivative with 24 being DDM [9] and the 2 others being Cymal-6 [48]. Consistent with observations by Psakis *et al*. [7], this might suggest that AcrB contamination and accidental crystallization is only problematic when using maltose-based detergents.

**Figure 6:**
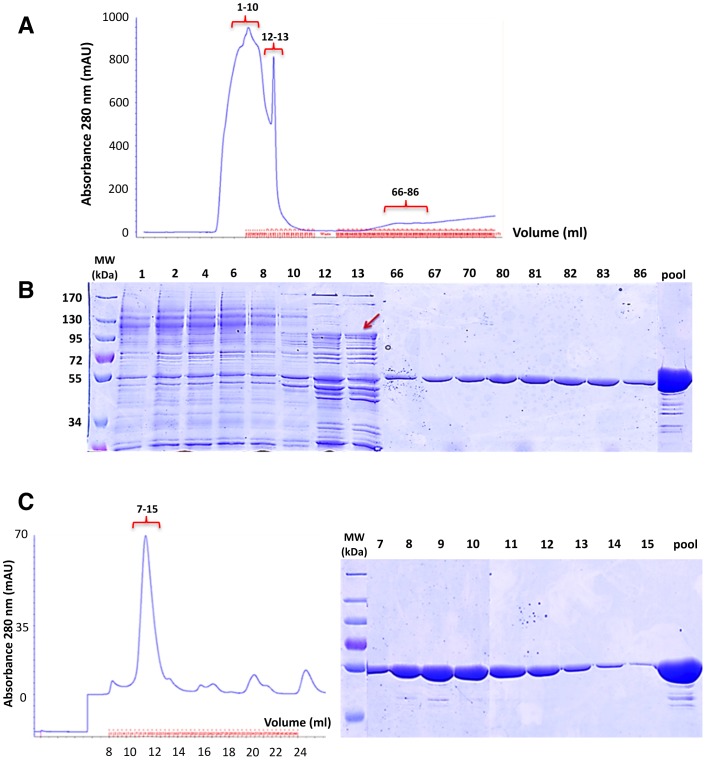
Strategy to eliminate the AcrB contamination. ***A***, chromatogram of DDM-solubilized BmrA eluted from a Ni^2+^-High Trap chelating column. The absorbance of the protein was monitored at 280 nm. The column was equilibrated and washed with 50 mM imidazole. ***B***, purification of BmrA was analysed by 10% SDS-PAGE. Fractions 1–10: Proteins loaded on Ni^2+^-High Trap chelating column, 12–13: BmrA washed with 50 mM imidazole, 66–86: BmrA eluted by linear imidazole gradient from 50 to 250 mM. The Red arrow indicates the position of AcrB. ***C***, left panel, size exclusion chromatography of BmrA loaded onto a Superdex 200 10/300 GL column. Right panel, the fractions 7–15 collected from the size exclusion chromatography were resolved on a 10% SDS-PAGE.

**Figure 7:**
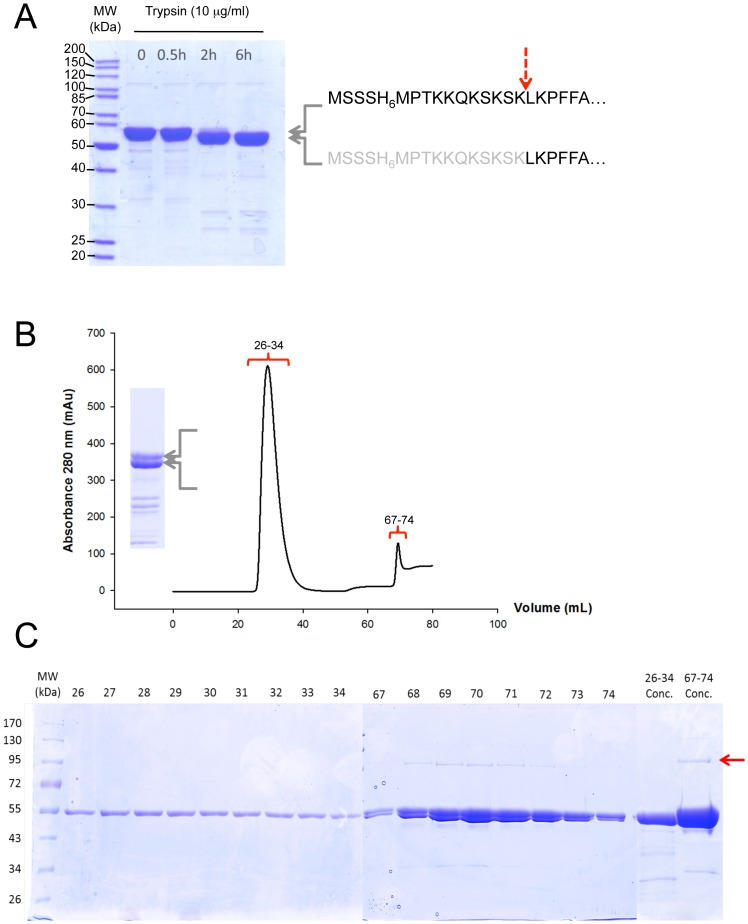
The cleavage of the His_6_-tag at the *N*-terminus of BmrA allows the elimination of AcrB by a second Ni^2+^–NTA chromatographic step. *A*, The E504Q BmrA mutant was incubated with 2 mM ATP and MgCl_2_ during 30 min 23°C, and trypsin (10 µg/mL) was added. At the time indicated, an aliquot was withdrawn, mixed with the SDS-PAGE loading buffer and kept on ice before being submitted to electrophoresis. After 6 h of incubation with trypsin, the ‘lower’ band migrating just below the full-length BmrA was identified by Edman sequencing and shown to correspond to BmrA lacking its *N*-terminal extremity and starting at Leu(12)-Lys(13)-Pro(14)-Phe(15)-Phe(16)… Therefore the main cut with trypsin occurred between Lys(11) and Leu(12) and is indicated by a red dashed arrow. *B*, After solubilization with DDM of membrane containing overexpressed E504Q BmrA mutant, extracted and non-extracted materials were separated by an ultracentrifugation and the supernatant was loaded onto a Ni^2+^ high trap chelating column followed by a PD-10 desalting column. The recovered E504Q BmrA mutant was incubated with 2 mM ATP and MgCl_2_ during 30 min and was submitted to trypsin digestion as in *A*. The incubation time was chosen to clearly see both the full-length, uncut protein, and BmrA with its *N*-terminal being cleaved (inset). The mixture was then submitted to a second Ni^2+^-High Trap chelating column as before and the chromatogram of the protein eluted from the column and monitored at 280 nm is shown. The first peak, fractions 26–34, corresponded to the unbound proteins and the second peak, fractions 67–74, to the proteins eluted with 250 mM imidazole. *C*, the different fractions obtained in *B* were analyzed by 10% SDS-PAGE. The two last lanes correspond to fractions 26–34 and 83–90 which were pooled separately and concentrated on a centricon (MWCO 50 kDa) before being submitted to electrophoresis. The Red arrow indicates the position of AcrB.

We developed three strategies in order to eliminate AcrB contamination. During DDM purification, using 50 mM imidazole in loading and washing buffers allowed to eliminate AcrB from the BmrA fractions (Fig. 6***A***). The gel filtration profile shows that BmrA was eluted in a homogeneous peak. SDS-PAGE confirmed the absence of AcrB in the pool, however we noticed the occurrence of some band below the main BmrA band at 55 kDa, perhaps due to some degradation. Alternatively, washing with FC12 could also be another possible solution for removing AcrB contamination from Ni^2+^–NTA resin during purifications. The second option would be to use a cleavable His-tag version of BmrA and to run a second Ni^2+^–NTA column to bind only the AcrB contaminant. Here, to avoid the cloning of BmrA in another vector with a cleavable tag, we took advantage of an observation we made when limited proteolyis experiment were conducted. Using trypsin, we were able to show that in a closed conformation of the transporter, namely using ATP/Mg plus vanadate for wild-type BmrA or ATP/Mg for an inactive E504Q mutant [34], it is possible to remove rather selectively the *N*-terminus of BmrA including the His-tag (Fig. 7*A*). Hence, after this cleavage by trypsin, the sequence of BmrA starts with L_12_KPFFA… When a short digestion with trypsin is performed on purpose, both the full-length and the untagged, cleaved, BmrA are resolved on a SDS gel (Fig. 7*B*). Then, running a second Ni^2+^–NTA column allows to recover the untagged BmrA in the flow through while the second peak eluted with imidazole contains a mixture of cleaved and full-length BmrA. This is very likely due to the fact that BmrA is a functional homodimer and part of these dimers in the second fraction contains a tagged, uncleaved, monomer and an untagged, cleaved, monomer. The pooled concentrated fractions show that in the first peak, containing untagged BmrA only, the AcrB contaminant is not present and it is only detected in the second peak eluted in the presence of imidazole (Fig. 7*C*). So this general approach with a cleavable tag should be useful to get rid of some of the contaminants that naturally bind to Ni^2+^–NTA column.

The third simplest strategy would be to use, whenever possible, a Δ*acrB E. coli* strain for the overexpression. This has been done here for BmrA using a BL21(DE3)Δ*acrAB*, Δ*acrEF* strain [49]. After solubilization with DDM, BmrA was purified by nickel affinity column and gel filtration and SDS-PAGE bands were analyzed by mass spectroscopy (Fig. 5***B***). Accordingly, the band 27 (∼110 kDa) shows the presence of two minor contaminants: pyruvate dehydrogenase and 2-oxoglutarate dehydrogenase.

**Figure 8:**
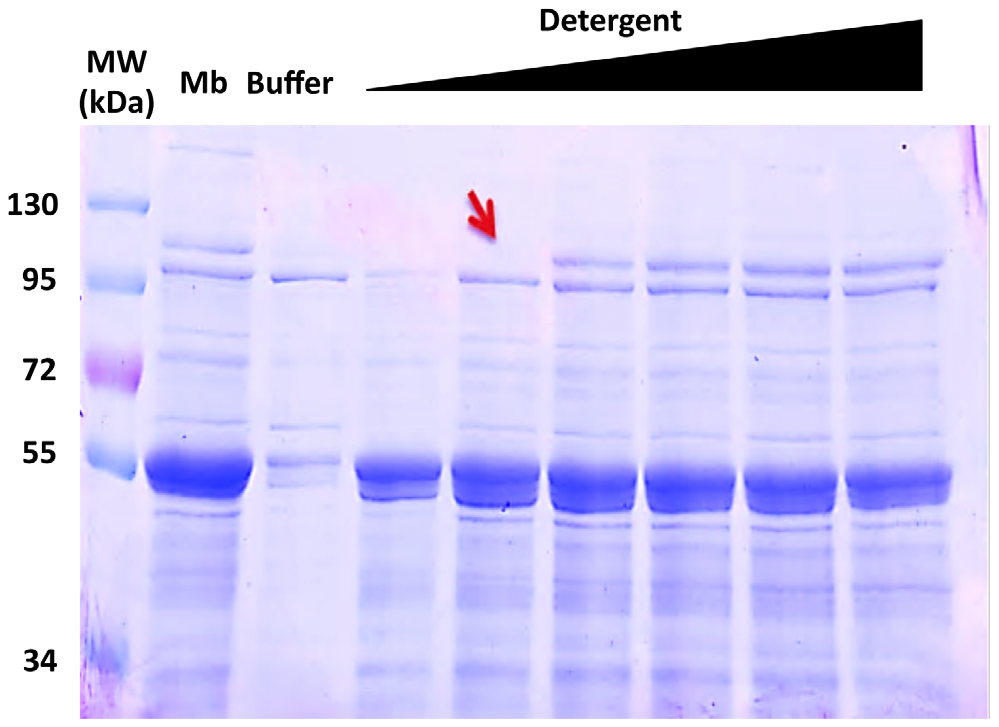
Strategy to eliminate the OmpF contamination. Extraction of BmrA using increasing concentrations of FC12. The concentrations of FC12 used were, 0.24% (5×CMC), 0.5%, 0.9%, 1.4%, 2.4% and 3.3% (70×CMC). Extracted material was resolved on a 10% SDS-PAGE at equivalent protein concentrations. Mb: the membrane fraction, Buffer: control without detergent. The Red arrow indicates the position of OmpF which is expected to migrate as a trimer in the conditions used for the electrophoresis [46].

Similarly, despite an increase in expression of OmpF during overexpression of recombinant proteins in *E. coli* [10], out of the tested detergents OmpF was only detected in the FC12 purified sample. Given the efficiency of FC12 membrane solubilization, this is perhaps not surprising. However, unlike AcrB there is no histidine-rich cluster present in OmpF making the level of contamination a bit surprising. Even the addition of a Q-Sepharose column (GE Heathcare) step to the purification protocol was unsuccessful in removing OmpF contamination (not shown). There are 17 previously reported structures of OmpF with 14 being with n-Octyl-oligo-oxyethylene (octyl-POE), 1 with n-Octyl-β-D-Glucoside (β-OG), and 2 with FC12 [10], but none with maltosides. This could again suggest that, like AcrB contamination, washing the Ni^2+^–NTA resin with a maltose-based detergent might be a possible solution for removing OmpF contamination. Here, we tested the extraction of BmrA with varying concentrations of FC-12 from 0.24% (5×CMC) to 3.3% (70×CMC; Fig. 8). In contrast to higher ratios, lower ratios of FC-12 did not solubilize OmpF because its band at ∼110 kDa was not detected anymore.

It should be noted that although OmpF was not detected in the maltosides or LDAO samples and AcrB was not detected in FC12 or LDAO samples we cannot rule out their presence. Veesler *et al*. [9] reported that AcrB was not detected by classical Coomassie-stained SDS–PAGE despite its accidental crystallization in attempts to crystallize the CorA transport protein. Similarly Psakis *et al*. [7] estimated that 100–500 pg of AcrB contamination in the crystallization drop was enough for crystallization. Therefore the only guaranteed method for an AcrB/OmpF free purified sample would be to start with overexpression using Δ*acrB* and Δ*ompF* knockout stains.

### Lauryl maltose neopentyl glycol

Crystallization trials are usually performed on samples of the highest purity to firstly increase the probability of obtaining initial crystals and secondly to obtain well-ordered crystals that diffract strongly as even trace contaminant can have detrimental effects [50]. Although there has been a report of crystallization of a protein out of a mixture of over fifty without any predominating species [51], the probability of crystallizing the target protein in such a mixture would be extremely low.

An encouraging new class of amphiphiles that are based on the maltosides known as the maltose-neopentyl glycols, exemplified here by LMNG, has recently been developed [52] and have been shown to dramatically increase the stability of membrane proteins compared to traditional detergents. Also, it has been reported that detergent exchange into LMNG from DDM generated large enough crystals in the lipidic-cubic phase of two new forms of the GPCR β_2_AR-T4L suitable for high-resolution diffraction while crystals were previously too small in DDM alone [52]. Similarly, three distinct conformations of β_2_AR were observed by NMR in LMNG that could not be seen in DDM [53]. Remarkably, unlike the other maltosides, mass spectral analysis of the band at 55 kDa of BmrA solubilized and purified with LMNG detected only BmrA. The only contaminating protein detected was unsurprisingly AcrB in the 110 kDa band (Fig. 5, band 8 and table 1). This is very encouraging as purifications starting from enriched membranes of BmrA over-expressed in a *ΔacrAB* strain would probably yield extremely pure protein. In addition to that, we compared the stability of BmrA in LMNG and in DDM. As shown in S1 Figure, almost no aggregates are detected in the LMNG purified sample after 6 days at room temperature in contrast to DDM where a significant amount of aggregates is observed just after 3 days of storage. Together with the previous reports of enhanced stability and diffraction the results here further support the use of LMNG in membrane protein crystallization of ABC transporters. Producing highly pure and stable protein is generally accepted to be the two most important parameters in successful crystallization, and purifications in LMNG possess both of these qualities.

## Conclusions

Detergents play a pivotal role in the solubilization, purification, and crystallization of membrane proteins. Our findings show that the relative amount and identity of contaminating proteins in purifications are strongly influenced by the detergent. Despite the use of affinity columns and SEC multiple contaminating proteins were detected and often had a similar migration pattern than the target protein making their initial detection difficult. Despite purifications starting from the same *E. coli* background, AcrB contamination was detected in all the tested maltose-based detergents, but not in FC12 or LDAO. Similarly, OmpF contamination was detected in FC12 but not in the maltosides-based detergents or LDAO. Both proteins have been repeatedly crystallized by accident highlighting the difficulty in their detection and removal throughout the purification protocols. Finally, the new detergent LMNG produced a highly pure protein that contained only AcrB as a contaminant. This stresses the usefulness of strains lacking AcrB or OmpF (or both) for overexpression of any membrane protein in *E. coli* whenever possible, or the optimization of the purification to get rid of these two nasty contaminants. Combining the strategies outlined here with recently developed FSEC or MC-SEC methods [14]–[16] could create a powerful platform for detecting and eliminating trace contaminates as well as assessing the stability, homogeneity, and stoichiometry in a fast and cost effective way for selecting only membrane protein samples of the highest quality possible to begin crystallization trials with.

## Supporting Information

S1 Figure **Stability of BmrA in DDM (** ***A*** **) versus LMNG (** ***B*** **).** Day 0 represents the initial SEC traces after the nickel elution (left Y-axis, blue traces). For days 3 and 6 the peak from day 0 corresponding to the BmrA dimer was pooled and concentrated to 10 mg/mL and incubated at room temperature to simulate crystallization conditions and re-injected after 3 days (DDM) and 6 days (LMNG) into SEC to monitor aggregation in the two detergents (right Y-axis, green traces). Please note that panels *A* and *B* were obtained using two different SEC columns: Superdex 200 10/300 (in ***A***) and Superdex 75 10/300 (in ***B***).
(PDF)

S1 Table **Data collection and refinement statistics.** Values in parenthesis are for the highest resolution shell. *R_merge_* = S_hkl_S_i_(I_i_<I>)/S_hkl_S_i_I_i_, where I_i_ is the ith reflection hkl and <I> is its mean intensity.
(DOCX)

S2 Table **Identification of the proteins present in the crystals loaded on SDS-PAGE.** Identification of the band migrating to ∼ 70 kDa by mass spectrometry reveals mainly the presence of OmpF in the crystals.
(DOCX)
