# Supplementary figures and images for: Stubborn Contaminants: Influence of Detergents on the Purity of the Multidrug ABC Transporter BmrA
Source: PLoS One. 2014 Dec 17;9(12):e114864. doi: 10.1371/journal.pone.0114864 (PMC4269414; doi:10.1371/journal.pone.0114864)

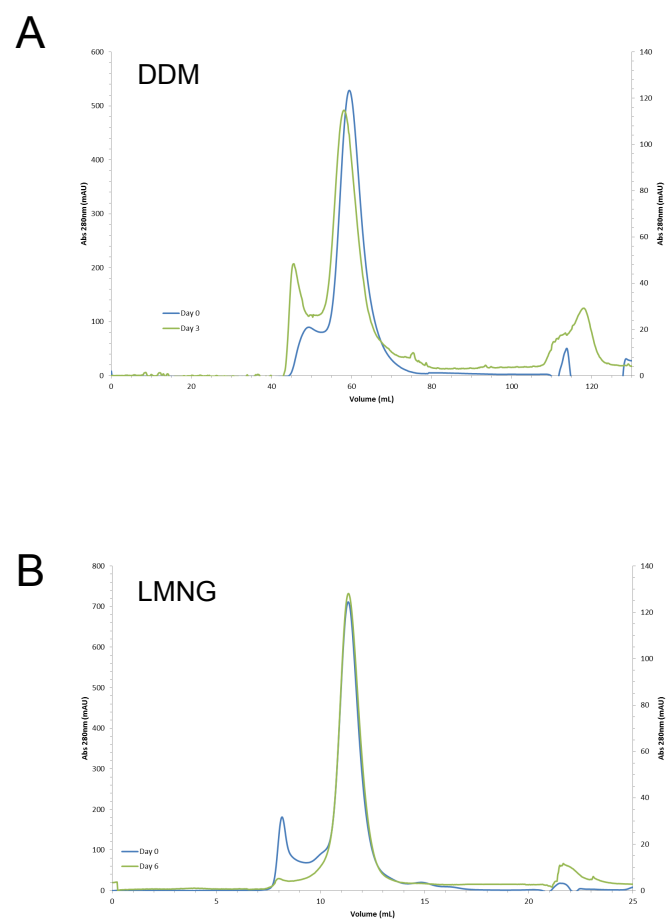

**Figure S1.**

Supplement: S1 Figure — Stability of BmrA in DDM ( A ) versus LMNG ( B ). Day 0 represents the initial SEC traces after the nickel elution (left Y-axis, blue traces). For days 3 and 6 the peak from day 0 corresponding to the BmrA dimer was pooled and concentrated to 10 mg/mL and incubated at room temperature to simulate crystallization conditions and re-injected after 3 days (DDM) and 6 days (LMNG) into SEC to monitor aggregation in the two detergents (right Y-axis, green traces). Please note that panels A and B were obtained using two different SEC columns: Superdex 200 10/300 (in A) and Superdex 75 10/300 (in B). (PDF) [file pone.0114864.s001.pdf]
