# Supplementary material for: Stubborn Contaminants: Influence of Detergents on the Purity of the Multidrug ABC Transporter BmrA
Source: PLoS One. 2014 Dec 17;9(12):e114864. doi: 10.1371/journal.pone.0114864 (PMC4269414; doi:10.1371/journal.pone.0114864)
Supplement: S1 Table — Data collection and refinement statistics. Values in parenthesis are for the highest resolution shell. Rmerge = ShklSi(Ii)/ShklSiIi, where Ii is the ith reflection hkl and is its mean intensity. (DOCX) [file pone.0114864.s002.docx]

| PDB code | **4JFB** |
| --- | --- |
| Space group | *C* 2 |
| Unit cell parameters (Å) | a=161.9, b=110.9, c=226.1, β=104.5° |
| Solvent content /ASU (%) | 71.3 |
| Resolution (Å) | 89-3.8 (3.97-3.8) |
| *R_merge_* (%) | 13.6 (49.9) |
| I/σ | 4.3 (1.6) |
| Completeness (%) | 96.9 (96.7) |
| Multiplicity | 2.8 (2.8) |
| Refinement | |
| N^o^. of reflections | 32027 |
| N^o^. of residues | 2040 |
| *R_work_* / *R_free_* (%) | 26.2 / 31.4 |
| Rmsd Bond Length (Å) | 0.006 |
| Rmsd Bond Angles (°) | 1.097 |

**Table S1**.
