# Supplementary material for: Stubborn Contaminants: Influence of Detergents on the Purity of the Multidrug ABC Transporter BmrA
Source: PLoS One. 2014 Dec 17;9(12):e114864. doi: 10.1371/journal.pone.0114864 (PMC4269414; doi:10.1371/journal.pone.0114864)
Supplement: S2 Table — Identification of the proteins present in the crystals loaded on SDS-PAGE. Identification of the band migrating to ∼ 70 kDa by mass spectrometry reveals mainly the presence of OmpF in the crystals. (DOCX) [file pone.0114864.s003.docx]

| **Score** | **# of**  **Peptides** | **Coverage**  **(%)** | **MW**  **(kDa)** | **Accession**  **#** | **Description** |
| --- | --- | --- | --- | --- | --- |
| 20121 | 1125 | 89,50 | 39,3 | P02931 | **Outer membrane protein F** |
| 133 | 5 | 18,27 | 43,3 | A7ZSL4 | Elongation factor Tu 1 |
| 105 | 3 | 2,21 | 64,5 | O06967 | BmrA |

**Table S2.**
